# Highly active antiretroviral therapy: Does it *Sound toxic*?

**DOI:** 10.4103/0975-7406.76494

**Published:** 2011

**Authors:** Katijah Khoza-Shangase

**Affiliations:** Department of Speech Pathology and Audiology, School of Human and Community Development, University of the Witwatersrand, Johannesburg, South Africa

**Keywords:** Adults, HAART, HIV / AIDS, monitoring, ototoxicity

## Abstract

**Objective:**

The main objective of the current study is to monitor the auditory status in a group of adults with AIDS, receiving Highly Active Antiretroviral Therapy (HAART) (3TC -lamivudine, D4T – stavudine, and efavirenz) in a hospital outpatient clinic in Gauteng. A total sample of 54 adults (between the ages of 18 and 50 years) in the experimental group and 16 in the control group were assessed prospectively following a repeated measures design. All participants were assessed at baseline at three months, and at six months into the treatment.

**Materials and Methods:**

The participants underwent case history interviews and medical record reviews, otoscopy, and tympanometry, as well as conventional pure tone audiometry and distortion product otoacoustic emission testing. Both descriptive and inferential statistics were used to analyze the data.

**Results:**

On audiological monitoring, statistically significant changes (*P*<0.05) were established, only in the experimental group, for pure tone audiometry — with clinically significant changes found at high frequencies. Statistically significant changes with clinically significant changes were obtained for distortion product otoacoustic emissions (DPOAEs) in the experimental group, particularly at high frequencies — implying subclinical hearing function changes; while lack of statistically significant changes with no clinically significant changes were found in the control group. The subclinical hearing changes in the experimental group were also evident in the findings of the subclinical hearing loss group, who, although they had normal pure tone function after six months of follow up, presented with clinical changes on DPOAEs at 6 and 8 kHz.

**Conclusions:**

Findings highlight the need for closer monitoring of the effects of antiretroviral drugs (ARVs) on hearing, through the use of more sensitive tools of assessment when conducting drug trials.

During the early stages of the human immunodeficiency virus / acquired immune deficiency syndrome (HIV / AIDS) pandemic, treatment strategies did not seem to have a positive influence on patients’ lives, and therefore, hearing loss did not seem to be an important manifestation of HIV / AIDS that required characterization. However, hearing loss has become one of a number of sensory disabilities associated with HIV / AIDS that must now compete for attention by the research and medical community. Friedman and Noffsinger[1] were among the first to advocate that as primary professionals in hearing healthcare, audiologists have a responsibility to inform both themselves and other relevant healthcare professionals about this issue, hence the current study.

Understanding the effects of HIV / AIDS and the treatment of HIV / AIDS on the auditory system is becoming more important, because patients with HIV / AIDS are living longer due to the positive effects of highly active antiretroviral therapy (HAART). The discovery of antiretroviral drugs for the treatment of HIV / AIDS has changed the face of the HIV / AIDS pandemic internationally, and has also led to changes in the medical field, with people who have HIV / AIDS living for longer periods of time experiencing toxic-related morbidity that influences the quality of life indicators.[2] There is a concern, however, that HIV-associated auditory disorders may be seriously underreported. Zuninga[3] makes a reference to anecdotal reports suggesting that hearing loss and dizziness, which are often the initial symptoms of the underlying auditory system disease may not have been reported by patients prior to HAART because many patients focused on the life-threatening complications of the HIV disease rather than on the quality of life issues. This situation is yet to be fully realized in developing countries, such as South Africa, where ARVs have not been available for a long time; and where available, access is not the same for the entire population infected by the virus. People who will benefit from these drugs in these countries may in the near future become more conscious of the quality of life issues and complain about them.

Numerous clinical, and mostly medically oriented studies have demonstrated the occurrence of hearing loss and other auditory manifestations in HIV / AIDS. Auditory manifestations may be one of the issues that the population will have to deal with; therefore, over and above the management of the known side effects of ARVs, research into the identification and monitoring of all other manifestations of the disease is required. According to the research literature, auditory abnormalities associated with HIV / AIDS and their treatment has been reported in persons with varying degrees of HIV infection, in both symptomatic and asymptomatic patients, as well as in patients on antiretroviral treatment. Indications exist that the HIV effects on the auditory system can be direct as well as indirect; however, this distinction is not always clear and consistent. Early reports in the literature demonstrated that the HIV might directly affect the auditory function due to the fact that the virus is neurotropic and commonly manifests itself neurologically,[4] which may be what Kallail *et al*.[5] term primary causes of auditory system disorders in HIV / AIDS. These direct causes have been reported to possibly give rise to the central pathology observed in this population.[6,7] More commonly, however, reports in the literature focus significantly on the indirect effects of the virus on the ear. It is believed that indirect causes that result in hearing loss stem from opportunistic infections that require suppressive therapy, thereby leading to ototoxicity;[6–8] which Kallail *et al*.,[5] refer to as iatrogenic sources. It is important to note that these findings are mainly from developed countries where the presentation and management of HIV / AIDS is different from that in the developing countries, suggesting a need for more research in this area, particularly as the number of adults living with HIV / AIDS in developing countries such as South Africa is still high, and also because the context is different. With regard to auditory manifestations, both identification and monitoring of ototoxicity require rigorous research, to enhance the patients’ quality of life, particularly as internationally, a link has been established between ARVs and ototoxicity.

On account of all the diseases and infections that the population with HIV / AIDS present with, it is not surprising to find patients with hearing loss due to ototoxicity, as this population goes through a drug regimen that often involves potentially ototoxic medications.[9] Bankaitis and Schountz[8] report that the use of experimental antiretroviral drugs, with undocumented or unknown side effects, contributes to this hearing loss. In addition, ototoxic drugs that are often used in the treatment of opportunistic infections such as tuberculosis may increase the potential for a drug-induced hearing loss in this population.[10]

Internationally, iatrogenic hearing loss has been associated with many of the drugs used to treat HIV / AIDS and its associated complications. As early as 1998, the potential for a drug-induced hearing loss in an HIV-infected individual at any stage of the disease was reported to be relatively high.[8] With all the medications that individuals with HIV are taking and the continual developments in HIV therapies, it is challenging to acquire and maintain a comprehensive knowledge base of HIV-related drugs and their associated ototoxicities. Although the side-effects of many antiretroviral drugs are yet to be determined, HIV-infected individuals are often prescribed medications as a prophylaxis or treatment of opportunistic infections that have been long associated with the development of audiological and vestibular changes. Antineoplastic medications such as vincristine, antifungal agents, including amphotericin B, flucytosine and ketoconazole, immune modulators, aminoglycoside antibiotics, erythromycin, and azidothymidine (AZT) are all widely used in the management of HIV and are all reported to be associated with significant ototoxicity or decreased hearing.[7,8,11–14] These medications are associated with hearing loss, tinnitus, and vertigo. Frequently administered medications for *Pneumocystis* pneumonia (PCP) (Pentamidine, TMP / SMX, Primaquine) may cause tinnitus, vertigo, dizziness, auditory disturbances, deafness, decreased hearing, hearing loss, and otalgia.[8] Moreover, the use of experimental medications with relatively unknown toxicity as well as the use of ototoxic drugs such as anti-tuberculosis (TB) medications, in combination, adds to the overall effect on hearing.[15]

In South Africa, one of the most frequently administered treatments for the HIV / AIDS population was TB treatment. South Africa, like many sub-Saharan countries, witnessed a dramatic upsurge of TB cases over the past decade.[16] This upsurge in the number of TB cases was expected to continue, largely due to co-infection with HIV, with the emergence of drug-resistant TB[17] also being reported. This co-occurrence of HIV/AIDS and TB raises serious implications for the audiologist with regard to the possible association between TB treatment and antiretroviral therapy (ART). As some of the drugs used in the treatment of TB fall under the umbrella term ‘aminoglycosides’,[18] interactions between these treatments need to be explored. Examples of these aminoglycosides include amikacin, gentamicin, kanamycin, netimicin, paromomycin, streptomycin, tobramycin, and apramycin.[19] These antibiotics are most notorious for being ototoxic, primarily targeting the renal and cochleovestibular system.[12] This impact of medications on the hearing function are being reported, although not extensively, with nucleoside analogue reverse transcriptase inhibitors (NRTIs).

Although a variety of adverse effects have been attributed to treatment with NRTIs for HIV-1 infection, only a small number of cases of ototoxicity have been reported in literature. Simdon reported three subjects who experienced ototoxicity, all of whom were over the age of 45 and received combination ART with two-to-three NRTIs plus a non-nucleoside reverse transcriptase inhibitors (NNRTI) or a protease inhibitor (PI). All three subjects had prior hearing problems, prior exposure to occupational noise, and all developed significant tinnitus.[20] Clearly, the presence of these confounding variables (prior hearing loss, noise exposure history, and older age) needs to be taken into consideration when interpreting the findings from these cases. The authors suggest that NRTIs must be used cautiously in patients with pre-existing hearing loss. Again, the ability to generalize these results is limited as they are based on case reports and not on large samples. These authors suggest that reductions in mitochondrial DNA content induced by NRTIs, as well as mitochondrial DNA mutations associated with aging and HIV-1 infection, may all contribute to auditory dysfunction in older patients with HIV-1 infection. They highlight the fact that prospective studies are necessary to determine the incidence of tinnitus and hearing loss among HIV-1-infected patients and their relation to the use of NRTIs.[20]

Several cases of ototoxicity have been reported in HIV-infected patients treated with zalcitabine;[21–23] didanosine;[24] zidovudine;[20] and combinations of zidovudine and didanosine;[25] stavudine and lamivudine;[15] stavudine, lamivudine, didanosine, and hydroxyurea;[15] and post exposure prophylaxis with stavudine, lamivudine, and nevirapine.[26] Moreover, a study of 99 HIV-infected individuals who received antiretroviral drugs showed that hearing loss was common in this population. Hearing loss was significantly associated with being 35 or older and with a history of ear infection, and there was a trend toward an association with a documented receipt of therapy with antiretroviral drugs in the preceding six months.[27]

As illustrated earlier, previous cross-sectional studies and case reports have shown an association between hearing loss and NRTI therapy.[15,27,28] There have been two case reports of hearing loss in persons receiving ART regimens that included NRTIs and a second class of antiretroviral drugs; one with an NNRTI (Nevirapine) and one with a PI (lopinavir / ritonavir), each combined with NRTIs, (both these subjects also received stavudine and lamivudine). One case reported sudden hearing loss two weeks subsequent to the person completing one month of post-exposure prophylaxis, which resulted in long-term hearing loss.[26] The other case described hearing loss in a subject with extensive HIV pre-treatment, which suggested a possible relationship with the protease inhibitor, although there were other possible explanations noted in Simdon’s reply to this case report.[20,29]

One should note that not all of the aforementioned studies utilized sensitive ototoxicity monitoring protocols such as ultra-high frequency audiometry and / or otoacoustic emissions. Furthermore, some of these studies did not follow longitudinal research designs either, which could have allowed the researchers to investigate within-subject changes; but they rather followed the cross-sectional methodology designs. In addition, the reports that other factors such as age, drug interactions, concomitant noise exposure, and so on may have an influence on the ototoxicity of ARVs should be taken into consideration when reviewing the effects of ARVs on hearing.

Although ototoxic hearing loss has been described in HIV-infected people after beginning NRTIs, there have been extremely limited prospective studies, with one published example of a prospective study by Schouten, Lockhart, Rees, Collier, and Marra.[30] Hence, there still need to be extensive investigations to clearly establish and confirm this relationship. The study by Schouten *et al*.[30] investigated hearing changes longitudinally in treatment-naïve HIV-infected subjects, following initiation of regimens containing NRTIs. The goal of their study involved performing a prospective assessment of the contribution of zidovudine (ZVD) and didanosine (ddI) to hearing loss. Changes in hearing levels at all frequencies and in low and high frequency pure tone averages were measured at baseline, 16, and 32 weeks after initiating antiretroviral therapy.

In Schouten *et al.’s*[30] study, treatment with ZVD and ddI did not result in loss of hearing, even after taking into account noise exposure, immune status, and age. The results of this prospective pilot study did not support the view that treatment with nucleoside antiretroviral drugs, damages hearing. This finding contradicts reports from previous cross-sectional studies and case reports that have indicated that hearing loss may be common among HIV-infected people due to ototoxic drug therapy.[27,31] The results of the prospective study by Schouten *et al*.[30] did not corroborate this relationship and are consistent with the report from the Adult / Adolescent Spectrum of HIV Disease Project Group that demonstrated no association between hearing loss and drugs used in the treatment of HIV. Of note, however, the Adult / Adolescent Spectrum of HIV Disease Project Group study was centred on a retrospective chart review for International Classification of Diseases (ICD) - 9 coding for hearing loss and not on a formal audiometry.[28] This represents a significant weakness in the methodology for a study attempting to determine the ototoxic effects, which can be subclinical in nature, hence requiring sensitive audiological monitoring tools.

There are at least three criticisms that can be leveled against the aforementioned study by Schouten *et al*.[30] First, this study did not incorporate otoacoustic emissions (OAEs) as part of their monitoring battery, and this could have had a significant impact on their results, as OAEs have been shown to be sensitive to cochlear damage in ototoxicity monitoring. Second, only 33 participants were included in their study, a small sample size that significantly reduces the strength of the study in terms of the ability to generalize the findings. Moreover, a small sample size limits the power of this study to detect a difference and also limits the ability to accurately interpret the results. Third, there was no control group, although the researchers did acknowledge that this was a pilot study. To their credit, these authors’ pure tone testing included 12 kHz, which is an ultrahigh frequency. Ultrahigh frequencies have been reported to be finely tuned to the effect of damaging environmental factors such as noise and ototoxic drugs.[12]

Replication of studies, such as Schouten *et al.’s*[30] longitudinal study, in developing countries such as South Africa, may be challenging due to a number of factors. First, the nature of the HIV / AIDS disease and the population being studied may preclude complete control over the confounding variables that could have had an influence on the results such as interactions of ARVs with other therapies; especially traditional medicine in the form of ‘*ubhejane*,’ which has been reported to be in widespread use.[32] Although, the current researcher is of the opinion that isolating all the possibly contributing confounding variables may provide a more accurate answer, it may not necessarily provide a practical, relevant, and context-sensitive finding. Within the South African AIDS population for example, it may be impossible to find participants who are only exposed to just one strict ARV regimen without any other medications coming into play. Second, securing a decent-sized comparison groups may be difficult, thereby preventing randomized matching of participants in the comparison group with those in the experimental group. Challenges in obtaining large enough sample sizes for control groups may be due to factors such as, attrition, on account of patients commencing treatment during the study as well as loss to follow-up. Third, ultra-high frequency audiometry, which does not form part of the routine audiological test battery, may influence the type of results found; and this may result in the clinical changes in the ultrahigh frequencies depicted on the audiogram being entirely missed. Finally, the length of time for which audiological monitoring occurred may be too short, due to attrition, to allow for the clinical hearing loss possibly caused by ART to manifest and therefore be detected on the audiogram.

Nevertheless, such longitudinal studies of patients on various regimens of ARVs need to be conducted. These need to be carried out in order to determine if any hearing changes occur during the period when the patients receive ARVs. Both clinically significant and statistically significant changes need to be investigated, as the presence of statistically significant changes does not necessarily translate to clinically significant findings. It is also critical that measures such as DPOAEs, which are sensitive to microcochlear changes, form part of the methodologies employed, as DPOAEs have been shown to be superior to pure tone audiometry in this regard;[33] hence the current study, which aims to monitor the auditory status in a group of adults with AIDS receiving HAART (lamivudine, stavudine, and efavirenz), in a hospital outpatient clinic, in Gauteng.

## Materials and Methods

### Research, aims, and objectives

#### Primary aim

The primary aim is to monitor the auditory status in a group of adults with AIDS receiving HAART (lamivudine, stavudine, and efavirenz), in a hospital outpatient clinic, in Gauteng.

#### Specific objectives

1. To longitudinally assess hearing function in AIDS-infected adults on HAART (experimental group);
2. To longitudinally assess hearing function in AIDS-infected adults not on HAART (comparison group);
3. To compare the results of the experimental group with those of the comparison group;
4. To analyze the hearing function in the group of adults with subclinical hearing loss.

The null hypothesis was that the participants’ hearing function before and after antiretroviral drug-use would remain the same. The alternative hypothesis was that it would not remain the same, that is, the participants would present with changes in their auditory function.[34–36]

### Design of the study

As an extensive literature search yielded a paucity of both South African and internationally published data on this topic, the study was exploratory and longitudinal in nature. The design utilized was a repeated measures, quasi-experimental design, with pre- and post-treatment testing, and a control group.[37] A quasi-experimental design was reported to be the best design where there were practical and ethical barriers to conducting randomized controlled trials.[38] In the current study, although there was manipulation of the independent variables (the antiretroviral drugs) and a control group, there was no random allocation of participants, which led to the study being quasi-experimental instead of experimental in nature.[34]

The antiretroviral medications and other therapies were the independent variables, with the audiological measures (otoscopy, impedance audiometry, pure tone audiometry, otoacoustic emissions) featuring as dependent variables. The aim was to investigate and monitor the auditory status in a group of adult patients with AIDS receiving ART and other therapies before and during antiretroviral treatment — with measures taken before commencement of ARVs, three months after initiation of treatment, and six months into therapy. All of the objectives were examined at baseline (before initiation of ARVs) and with repeated measures (three and six months into treatment) for both the control and experimental group. A comparison of results of the control group and experimental group was done for all objectives.

One methodological limitation of the current study was related to the time when the last measure was carried out (six months after treatment). This could have limited the type of results obtained, as ototoxicity could present long after six months; however, the researcher decided on this time in an attempt to control for variables such as: patients changing medications, participant attrition via patients leaving the study for various reasons, and so forth.

### Participants

A total sample of 54 adults (between the ages of 18 and 50 years) in the experimental group and 16 participants in the comparison group participated in the study.

The patients selected for this study were recruited from the Hospital’s Adult HIV / AIDS clinic. Patients attending this clinic had already been diagnosed with HIV / AIDS and were seen there for general medical management as well as antiretroviral treatment and monitoring. At the time of the study all patients with CD4+ counts below 200 cells / mm^3^ had access to ARV treatment at this clinic — and this was the group that was targeted for the experimental group.

### Participant selection criteria - Inclusion criteria

Given the fact that little, if any, published research had been conducted on this aspect of HIV/AIDS in South Africa, the researcher believed that it was crucial to have a high degree of control over the variables, which could confound the results of the study (e.g., noise exposure, syphilis, and so forth).[37,39] Consequently, the participant inclusion criteria that were adopted following baseline testing are depicted in Table 1.

**Table 1:** Summary of participant inclusion criteria following baseline measures

Criterion	Experimental group	Control group
HIV / AIDS positive serology	Yes	Yes
On ARVs	Yes	No
Age between 18 and 50 years	Yes	Yes
Alert and oriented	Yes	Yes
Normal pure tone audiometry (thresholds better or equal to 25 dBHL)	Yes	Yes

### Participant selection criteria - Exclusion criteria

The following criteria were strictly observed for persons who participated in the repeated measures following baseline [Table 2]:

**Table 2:** Summary of participant exclusion criteria following baseline measures

Criterion	Experimental group	Control group
Noise exposure	Yes	Yes
Recent (less than three years) or current history of treatment for TB and radiotherapy	Yes	Yes
Positive clinical or serological evidence of syphilis	Yes	Yes
Middle ear pathology	Yes	Yes
Presence of tinnitus	Yes	Yes
Recent (less than three years) history of previous ARV use	Yes	Yes

### Recruitment and sampling procedure

A nonprobability convenience sampling technique was utilized in the study, as the sample was restricted to a part of the population that was readily available,[37,39] and true random sampling would have been difficult to achieve due to time, cost, and equipment limitations.

Participants for the control group were recruited from the wellness section of this clinic, where patients who refused treatment were seen by Dieticians and Social Workers. At the time of the study, the researcher was already providing service to patients at this clinic, as she was on an honorary appointment as an Audiologist at the research site.

### Research procedures and materials

Participants underwent case history interviews and medical record reviews, otoscopy and tympanometry, as well as conventional pure tone audiometry and distortion product otoacoustic emission testing. Baseline data were collected by assessing the participants’ dependant variables before administration of ARV therapy. These baseline data were then compared with two other measures that were taken three and six months after commencement of therapy. The same procedures were followed for the control group. For those participants who did not attend all three sessions of testing, their data were excluded from the inferential statistical analysis, as this required data from all three sessions.

Following infection control measures proposed by Kemp and Roeser,[40] all testing was conducted in a sound-proof booth. Basic audiological testing followed by DPOAE measurements for all participants was undertaken and systematically recorded.

#### Case history

A case history form that targeted the signs and symptoms of auditory manifestations was utilized, in order to gather all the important case history information, audiological data, and some medical variables that could have exerted an impact on the results of the study.

#### Otoscopy

The researcher evaluated the participants’ ears for the presence of impacted wax; otitis externa; possible otitis media; perforated tympanic membranes; collapsed ear canals; presence of any growths, or any other ear disorders.[41] These otoscopic abnormalities were reported to have a significant effect on DPOAE and therefore needed to be documented before testing commenced.[33]

#### Impedance audiometry

Impedance audiometry in the form of tympanometry (through the use of Inter-AcousticAZ26 audiotympanometer) was utilized to assess the status and integrity of middle ear functioning. A standard single frequency tympanometry using an 85 dB SPL tone, set at 226 Hz was done. The primary purpose of impedance audiometry was to determine the status of the tympanic membrane and middle ear via tympanometry. The researcher ensured that all participants undergoing DPOAE testing had normal (type A tympanogram) tympanometry results.

#### Pure tone audiometry

Conventional (250 Hz – 8000 Hz) pure tone audiometry was performed on all participants through the use of an Inter-Acoustic AC 40 diagnostic audiometer. The criteria used to define normal hearing, was that of pure tone thresholds of 25 dBHL or lower across all frequencies, with the absence of an air-bone gap.[42] If pure tone air conduction and tympanometry were abnormal at any test frequencies, in the pre-treatment phase, the participants were excluded from continuing in the study, and were referred to the Ear, Nose, and Throat Specialists for assessment and management, and were subsequently offered appropriate audiological rehabilitation. Participants presenting with normal pure tone audiometry at the baseline were advanced to sessions two and three of the study, where ototoxicity monitoring was conducted. Using pure tone data, a change in the hearing level of 10 dB at one or more frequencies was commonly taken to be indicative of some significant change,[43] hence, this protocol was followed in the current study.

#### Distortion product otoacoustic emissions

All participants with normal middle ear functioning underwent DPOAE measurements, as a crucial aspect of ototoxicity monitoring, through the use of a Biologic Scout Otoacoustic emissions meter. OAE testing is often used as a screening tool to determine the presence or absence of cochlear function, and analysis can be performed for individual cochlear frequency regions, therefore, they are regarded as an excellent tool for the early detection of cochlea damage due to ototoxicity.[12,33] OAEs can detect cochlear dysfunction before it is evident on pure tone audiometry, and in ototoxicity monitoring this factor is critical, as the main aim of monitoring is the early detection of adverse effects of the drug before it causes clinical damage.[12,33,44] One methodological limitation of the current study was that the ultra-high-frequency audiometry did not form part of the ototoxicity monitoring protocol due to lack of equipment available at the time of the study. Ultra-high-frequency testing has been reported to be sensitive to ototoxicity. As far as DPOAEs are concerned, the current study only monitored frequencies up to 8000 Hz - and this was another acknowledged methodological limitation, as literature has indicated greater sensitivity when using higher frequencies in ototoxicity monitoring. The following DPOAE test protocol was employed:

**Table:** 

Test parameters	Diagnostic / High frequency
Stimuli	
Intensity level	L_1_-L_2_ = 10 dB (e.g., L_1_ = 65 dB, L_2_ = 55 dB)
Ratio	f_2_/f_1_ = 1.22
Frequency range	750 to 8000 Hz

The presence of the DPOAE was determined by comparing the amplitude of the DPOAE to that of the noise floor to calculate the size of the emission. A DP amplitude that exceeded the noise floor by at least 7 dB across all frequencies measured was regarded as indicative of a normally functioning cochlea.[33] The size of the emission at the different frequencies measured was then monitored over the three testing sessions.

### Validity and reliability

Test reliability was controlled and maintained at a high level by standardizing test administration, ensuring proper equipment calibration, and controlling patient variables. For all audiological assessments precautionary measures advocated by Bess and Humes[45] and Hall[33] were followed in terms of proper maintenance and calibration of the equipment; optimizing testing environment; correct earphone and bone vibrator placement, and proper probe placement for DPOAE. All testing was conducted in a soundproof booth or sound-treated room with equipment that was calibrated on an annual basis, with biological calibration conducted before every test session. All the participants were tested by the same researcher using the same test procedure at all three sessions. Furthermore, all the patients were tested in the mornings to reduce the effect that fatigue could have on the patients’ responses to behavioral audiometry testing.

However, threats to validity in the current study were present, and they included the fact that the study was not a double-blind study, as the researcher was aware of which participants were in the control group and which were in the experimental group. There was no random selection of participants to reduce bias in the sample; and there was limited control over the confounding variables such as interaction of ARVs with previous exposure to ototoxic drugs, and interaction of ARVs with other routine medications and supplements that were being prescribed at the time of the study.[34,38] Finally, due to the sample size and the fact that the data were collected in one hospital in Gauteng, South Africa, the researcher’s ability to generalize the results from the sample studied to the total population of adults with AIDS in South Africa is limited.

### Data analysis and statistical procedures

Both descriptive and inferential statistics were used to analyze data from the study. Inferential statistics in the form of repeated measures, using analysis of variance (ANOVA), Multivariate analysis of variance (MANOVA), and Tukey-Kramer post-test were used to establish statistical significance levels, and to determine when the statistically significant changes occurred within the longitudinal design of the study. Furthermore, the clinical significance of the findings was also analyzed.

First, a statistical comparison was done, basing the results on the average change from the baseline. Each frequency’s mean change from the baseline for the ears, individually, was computed and then combined for both pure tone audiometry and distortion product otoacoustic emissions. Repeated-measures analysis of variance[34] was used to compare the mean change from the baseline for the control group and the experimental group, from session to session. In the analysis of the DPOAE data, the baseline DPOAE levels in decibels SPL for each f2 value tested were compared with the corresponding measurements in sessions 2 and 3. The signal-to-noise difference was used as the measure of DPOAE amplitudes. For pure tone audiometry data, the baseline thresholds in decibels HL for each frequency were compared with the corresponding results in sessions 2 and 3 as well. To statistically test the hypothesis, a threshold *P* value (alpha) of 0,05 was selected.[46] Finally, as part of a statistical analysis of the data, a post-test in the form of the the Tukey-Kramer multiple-comparison post-test was conducted. This test was conducted to compare pairs of group means so as to identify where, precisely, statistically significant changes occurred along the time continuum (baseline to six months) - if they did.

Second, for the purposes of the current study, clinical significance (changes that are deemed significant enough to indicate structural and functional changes in the ear) over and above the statistical significance was examined. There is a growing recognition that assessing an intervention’s effect should not only focus on the statistical significance of the differences between the experimental and control groups, but should also focus on the relevance or importance of these outcomes.[47] For pure tone testing, most often a change of 10 dB at one or more frequencies is commonly taken to be indicative of some significant change;[43] and this is the protocol followed in the current study. As far as DPOAEs are concerned, change is only regarded as significant in the DPOAE measures if there is a change of at least more than 6 to 9 dB in the DPOAE level between consecutive measures.[48,49] Absence of clinically significant changes in the DPOAEs confirm intact cochlear functioning and the absence of cochlea damage, even microcochlear pathology - which is often evident on OAEs, long before being depicted on the pure tone audiogram.[33]

During exploratory data analysis, it was discovered that there were participants in the experimental group who presented with changes on DPOAE measures, without changes in pure tone audiometry. This presentation of results necessitated an additional analysis step where these participants (referred to as the *subclinical hearing loss group*) were analyzed separately; hence this formed an additional set of results.

The following table [Table 3] provides a summary of all collection material and test procedures used in this study.

**Table 3:** A summary table of collection materials and test procedures used in the current study

Equipment	Function	Pass criteria	Fail criteria
Case history form	Gather important case history data	Refer to inclusion and exclusion criteria	Refer to inclusion and exclusion criteria
Welch Allyn Otoscope	Visual inspection of the ear	Clear outer ear with normal tympanic membrane	Obstruction; abnormal tympanic membrane, pathologies of the outer and middle ear
AZ26 Interacoustic tympanometer	Middle ear functioning assessment	Type A tympanogram	Other tympanograms, but type A
AC40 Diagnostic audiometer	Conventional pure tone audiometry (250 – 8000 Hz) Monitoring function	Thresholds at and better than 25 dBHL and no Air-Bone Gap No 10 dB threshold change at one or more frequencies over time	Thresholds worse than 25 dBHL A change of 10 dB at one or more frequencies over time
Biologic Scout OAE machine	Diagnostic DPOAE measurement	Greater than 7 dB DP amplitude at frequencies assessed	Less than 7 dB DP amplitude
	Ototoxicity monitoring	No DPOAE level change of more than 6 to 9 dB between consecutive measures	Change of more than 6 to 9 dB in the DPOAE level between consecutive measures

### Ethical consideration

Prior to the commencement of the study, permission to conduct the research project was sought from the University of the Witwatersrand Human Research Ethics Committee (Medical), which gave unconditional ethical clearance in the form of protocol number M041131. The researchers ensured that permission to conduct the study was obtained from the hospital management and from the Heads of the Audiology and HIV / AIDS clinics at the research sites. Written informed consent to participate in the study was obtained from all the participants before the study was conducted, with an assurance that confidentiality of all records would be maintained. Furthermore, to ensure anonymity, the researcher ensured that no personal or identifying information was included in the research report and research coding numbers instead of identifying information were used. The current study also reduced risks to the participants to a minimum, by conforming to the ethical principles[50] and observing provisions of the Nuremberg Code of ethics[34] during the study. Finally, the hospital and participants could request to see the research results if they were interested.

## Results

**Figure 1:**
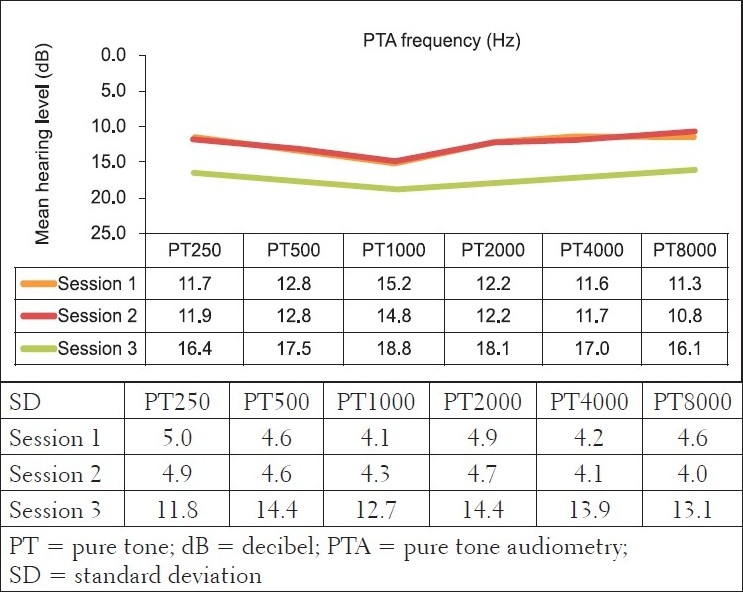
Mean bilateral pure tone audiometry results (in dBHL) and their standard deviations for the control group at the three different sessions (n=32 ears)

As indicated in Table 4, the current investigation revealed noteworthy findings. As the researcher looked at both the statistical and clinical significance, the findings highlighted the importance of including both these means of establishing the significance in any longitudinal audiological study. Specifically, pure tone audiometry results in the comparison group revealed hearing within normal limits, with the average PTAs being above the level regarded as indicative of normal hearing across all frequencies evaluated, as depicted in Figure 1. These mean results were normal for all three testing sessions. With regard to the MANOVA tables, all changes in the comparison group were found not to be statistically significant over the three testing sessions. Moreover, these changes were also not audiologically clinically significant changes. Most often a change of 10 dB at one or more frequencies is taken to be indicative of a clinically significant change.[43] None of the mean changes in pure tone results of the comparison group in the current study were greater than 10 dB; suggesting that they were not clinically significant changes.

**Table 4:** Summary of ototoxicity monitoring findings from the current study

Factor	Comparison group (N=16)	Experimental group (N=54)
Pure tone audiometry (PTA)	Normal throughout the three testing sessions	Normal throughout the three testing sessions
Clinical analysis	No clinically significant changes	No clinically significant changes
Statistical analysis	No statistically significant changes	Statistically significant changes at 8000 Hz
Distortion product otoacoustic emissions (DPOAEs)	Normal OAEs throughout the three testing sessions	Reduced / absent OAEs at session 3
Clinical analysis	No clinically significant changes	Clinically significant changes at 6 and 8000 Hz at session 3
Statistical analysis	No statistically significant changes	Statistically significant changes at session 3

Key: PTA change > 10 dB = Clinically significant change; DPOAE change > 6dB = Clinically significant change. *P* < .05 = statistically significant change

**Figure 2:**
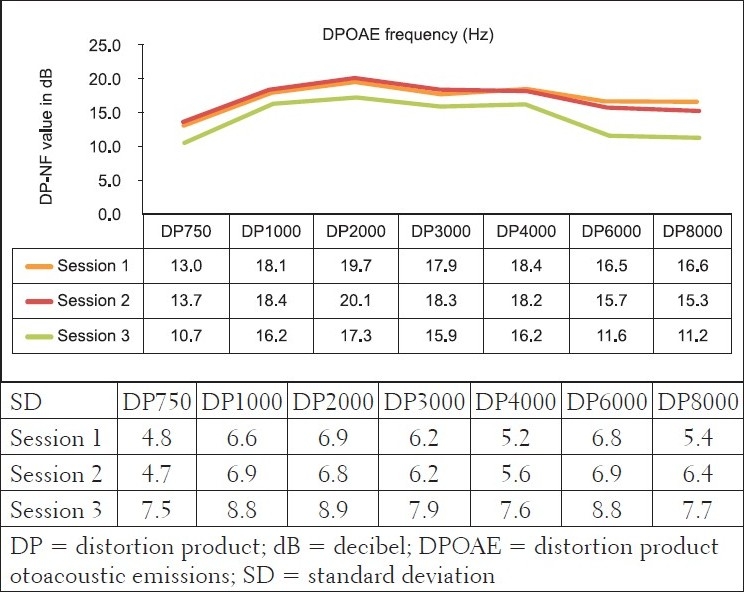
Mean bilateral distortion product otoacoustic emission results (in dBSPL) and their standard deviations for the control group at the three different sessions (n=32 ears)

Furthermore, DPOAE measures for the comparison group [Figure 2] revealed cochlea functioning to be within normal limits, with the average DPOAE emission size being above the level regarded as indicative of normally functioning cochleas, across all frequencies evaluated for all three sessions. The MANOVA tables indicated that changes were not statistically significant (*P*>.05). Furthermore, these changes were also not clinically significant. Change is only regarded as clinically significant in DPOAE measures if there is a change of at least 6 to 9 dB in the DPOAE level between consecutive measures.[48,49] In the comparison group, none of the mean changes in DPOAE results were greater than 6dB - indicating that no clinical changes occurred in the cochlear function. The absence of clinically significant changes in pure tone audiometry and DPOAEs in the comparison group, in the three different sessions, over a period of six months, confirmed intact hearing functioning and the absence of any evidence of cochlea damage, even microcochlear pathology - which is often evident in OAEs long before being depicted on the pure tone audiogram.[33]

**Figure 3:**
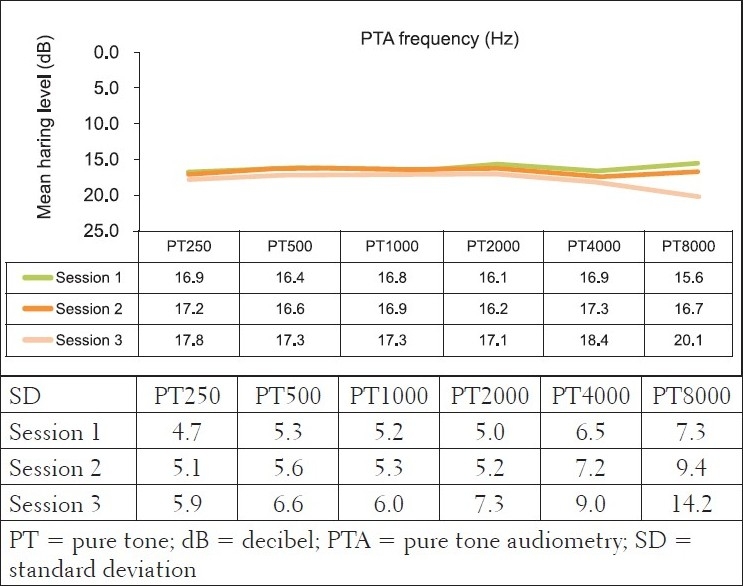
Mean bilateral Pure Tone audiometry results (in dBHL) and their standard deviations for the experimental group at the three different sessions (n=108 ears)

For the experimental group, however, the ototoxicity monitoring phase yielded different results to those of the comparison group, in that, some results indicated the presence of both statistically and clinically significant changes [Table 4]. For pure tone audiometry data, the criteria for clinically significant change were not met — none of the mean changes in the pure tone results were greater than 10 dB [Figure 3]. The MANOVA tables for repeated measures analysis of variance (within group) revealed no statistically significant changes (alpha was greater than 0.05), with the exception of significant changes at 8000 Hz [left ear (*P*=0.04)].

**Figure 4:**
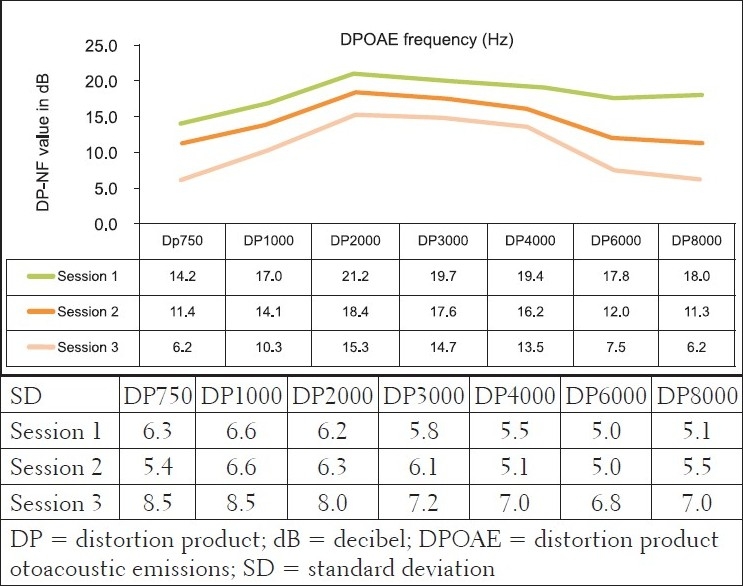
Mean bilateral DPOAE results (in dBSPL) and their standard deviations for the experimental group at the three different sessions (n=108 ears)

However, results of the DPOAE analysis revealed the cochlea function to be normal at sessions 1 and 2, at all frequencies evaluated, with changes at repeated measures, indicating declining DPOAE values [Figure 4]. These changes were found to occur at all evaluated frequencies, but were more clinically significant at 6 and 8 kHz bilaterally in session 3. The DPOAE results at these two frequencies at session 3 were in fact below the norm, in that, the DPOAE value did not exceed the noise floor by at least 7 dB, as expected in a normally functioning cochlea. Statistically, the MANOVA [within group (time)] results indicated extremely significant *P* value (*P*<.001) for all frequencies assessed - implying that the cochlear function changed after ARV initiation.

The presence of significant changes on DPOAEs in the three different sessions, over a period of six months, in the experimental group, indicated possible microcochlear pathology that was not necessarily indicated on pure tone audiometry. Hence, it would seem to be a possibility that based on the objective nature of the DPOAE measures, subclinical auditory changes occurred after a six-month period. This finding also highlights the crucial need for the use of such sensitive measures (DPOAE) in monitoring the possible effects of toxins on the ear, as DPOAEs have been shown to be superior to pure tone audiometry in this regard.

**Figure 5:**
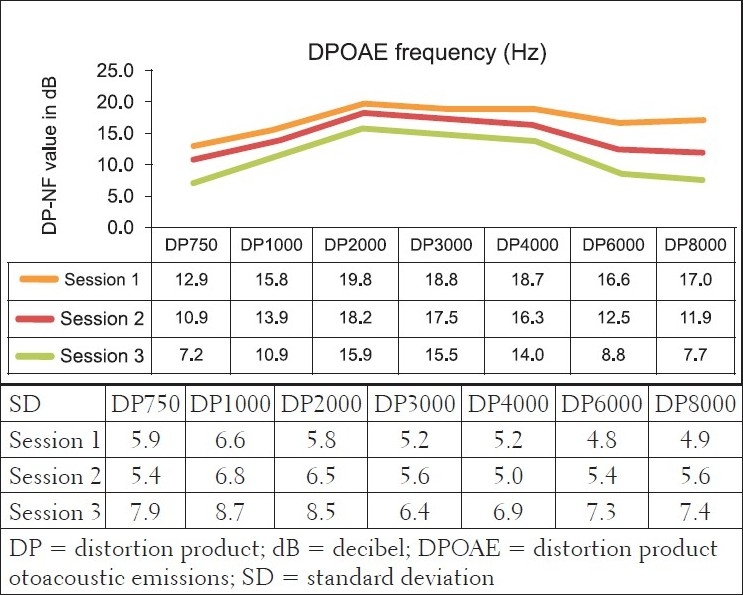
Mean bilateral DPOAE results (in dBSL) and their standard deviations for the group with normal PTA at the three different sessions (n=90 ears)

For the statistically significant results for DPOAEs in the ‘within group’ analysis, the Tukey-Kramer test indicated that generally, significant changes occurred between baseline measures and session 2 (at three months) for the lower frequencies, with the higher frequencies being significantly affected all through the three sessions of testing. This early onset of symptoms (although subclinical) again highlights the need for early involvement of the audiologist in the assessment and management of patients with AIDS. Findings from the *subclinical hearing loss* group further illustrate that subtle subclinical changes can only be identified by DPOAEs and not pure tone audiometry. Figure 5 depicts the findings of 45 participants who presented with normal pure tone audiometry results, but showed significant changes on DPOAEs over the three testing sessions.

The presence of DPOAE changes indicating subclinical changes in hearing status suggested hearing loss that could have possibly been due to medications used by the participants in the experimental group (lamivudine, stavudine, and efavirenz). These results were consistent with the reports that have associated iatrogenic hearing loss with many of the drugs used to treat HIV / AIDS.[11,14]

Although the current study may not be able to draw a definitive conclusion that ARVs used in the sample had a direct effect on hearing - because of the difficulty in controlling the other interacting factors - an index of suspicion is raised by the fact that the results of the participants in the experimental group seem more consistent with ototoxic hearing loss than those in the comparison group, and the fact is that the only identifiable difference between the two groups is that of ARV use. However, the current researcher is of the opinion that isolating all the possible contributing confounding variables may provide a more accurate answer, but may not necessarily provide a practical, relevant, and context-sensitive finding. Within the South African AIDS population, it may be impossible to find participants who are only exposed to just one strict ARV regimen without any other medications coming into play. The results of this study are felt to be valuable and applicable to the South African context, and may provide more realistic and context-specific implications; those that are needed for establishment and implementation of ototoxicity monitoring protocols as part of the routine clinical management of adults with HIV / AIDS.

## Discussion and Conclusion

This study displays and highlights an expanded role of the audiologist in Food and Drug Administration processes of drug development, drug approval, and drug monitoring. The sub-clinical hearing changes (cochlea function changes detected before the hearing loss is seen on an audiogram) highlight the importance of DPOAEs in ototoxicity monitoring studies.This research also demonstrates the important role that the audiologist may have in both the assessment and treatment of patients with HIV / AIDS. Finally, implications raised by the current study can be translated into recommendations for the clinical assessment and management of patients with HIV / AIDS who are taking ARVs; education of team members; policy formulation; as well as further research.

Medical awareness of ARV doses, forms of administration, populationsat risk, and possible synergism with other factors is necessary to developappropriate care in the prescription of drugs with possible or established ototoxicside effects. Furthermore, issues such as risk-benefitanalysis, patient-informed consent, and quality-of-life considerations, are also crucial factors to beconsidered in the management of patients with HIV / AIDS. Regardless of whether the effects of the drug are negligible or not, these effects still need to be determined so that proper patient adherence counseling can take place. It is fundamental that audiologists establish and become aware of the ototoxic effects of medications used to manage chronic conditions such as HIV / AIDS, as also medications prescribed to significant numbers of people - such as 11% of the population afflicted by HIV / AIDS in South Africa.[51] This awareness is critical, to ensure that proper patient education occurs, as patients may not notice ototoxic hearing loss until a communication problem becomes evident, signifying that hearing loss within the frequency range, which is vital for understanding speech, has already occurred. Likewise, by the time the patient complains of dizziness, permanent vestibular system damage may have already occurred.

Clinically used drugs and chemical agents may potentially causeadverse effects to the human auditory and vestibular systems.[52] Many of these drugs can playa critical role in the treatment of serious or life-threateningdiseases. others offersuch important therapeutic effects compared to the ototoxicside effects that the ototoxicity risk can be considered tobe of minor importance, and such may be the case with HIV / AIDS (a sentiment echoed by some physicians). The problem of ototoxic side effectsis reported to be more critical in developing countries, where highly effectiveand low-cost drugs are more easily prescribed without adequatemonitoring.[53] It is possible that such a situation may exist in some parts of Africa, particularly with the high numbers of patients on treatment for HIV / AIDS. An additional concern in the management of HIV / AIDS patients, who may be on potentially ototoxic medication without being audiologically monitored, is that noise exposure following treatment with ototoxic drugs can act synergistically with the drugs that have not been fully cleared from the inner ear.[54] Increased susceptibility to hearing loss can continue for several months after completion of treatment or therapy. Due to this likelihood, it is imperative to implement hearing conservation in the form of advising patients to avoid excessive noise exposure for at least six months. In addition, patients who use amplification in the form of hearing aids may need to be counseled and warned to closely monitor and control the hearing aid maximum output during this critical time.[55] Given this scenario, it seems more pressing than ever to endeavor to prevent or ameliorate the possible ototoxic hearing loss in this population, by ensuring ototoxicity monitoring as part of the routine clinical management; particularly as the treatment regimen is varied and the WHO ART guidelines continue to be modified, as some drugs get phased out, such as the recent suggestion by WHO ART to phase out d4T.

When life-threatening illness necessitates treatment with ototoxic drugs, preserving the quality of the patients’ remaining life is customarily a treatment goal. Early detection of ototoxic hearing loss provides physicians with critical information and the opportunity necessary to minimize further impairment, and in some cases, prevent hearing loss from progressing to the point where permanent damage occurs. Although hearing loss is not regarded as a life-threatening condition, it does become a severe threat to the essential quality of life indicators unless intervention occurs early during treatment. The adverse effects of hearing loss on cognitive-linguistic skills and psychosocial behavior are well documented, as also the serious vocational, social, and interpersonal consequences for the patient.

Findings of the current study, although important, should be interpreted after taking the identified methodological limitations into account. The main limitations of the current study included, first, the nature of the HIV / AIDS disease and the population being studied precluded complete control over the confounding variables, which could have had an influence on the results such as interactions of ARVs with other therapies; especially traditional medicine in the form of ‘*ubhejane*,’ which has been reported to be in widespread use.[32] Second, the sample size for the comparison group was small, thereby preventing randomized matching of participants in the comparison group with those in the experimental group. The small sample size of the comparison group was due to factors such as attrition, due to patients commencing treatment during the study, as well as loss to follow-up. Third, ultra-high frequency audiometry did not form part of the test battery and this may have influenced the type of results found, in that, clinical changes in the ultra-high frequencies depicted on the audiogram could have been missed. Finally, the length of time for which audiological monitoring occurred (six months) may have been too short to allow the clinical hearing loss, possibly caused by ART, to manifest, and therefore, be detected on the audiogram. Nevertheless, findings do justify an intense pursuit of the answer to the question: Does HAART *sound toxic?*

The known effects of HIV / AIDS on the auditory system that have been reported in the literature are mainly based on cross-sectional studies and case reports conducted internationally in industrialized countries, with very limited information coming from third world countries where the presentation of the virus and its treatments may be different. Furthermore, because this evidence may not be viewed to be contextually relevant to the developing world, its incorporation into routine clinical assessment and management lags behind significantly. Hence, the need for categorizing the ototoxic effects of HIV / AIDS treatment, in an effort to ensure that ototoxicity monitoring protocols are established and implemented as part of the routine clinical management among infected patients. Research in ototoxicity in HIV / AIDS needs to be locally relevant, should include large sample sizes and longitudinal follow-up of cases, and should also utilize sensitive audiological test measures to improve the validity and reliability of the findings.
